# Head-to-head comparison of BAM15, semaglutide, rosiglitazone, NEN, and calorie restriction on metabolic physiology in female *db/db* mice

**DOI:** 10.1016/j.bbadis.2023.166908

**Published:** 2023-10-02

**Authors:** Sing-Young Chen, Martina Beretta, Ellen M. Olzomer, Stephanie J. Alexopoulos, Divya P. Shah, Frances L. Byrne, Joseph M. Salamoun, Christopher J. Garcia, Greg C. Smith, Mark Larance, Andrew Philp, Nigel Turner, Webster L. Santos, James Cantley, Kyle L. Hoehn

**Affiliations:** a School of Biotechnology and Biomolecular Sciences, University of New South Wales, Sydney, NSW 2052, Australia; b Department of Chemistry and Virginia Tech Centre for Drug Discovery, Virginia Tech, Blacksburg, VA 24061, USA; c School of Medical Science, University of New South Wales, Sydney, NSW 2052, Australia; d Charles Perkins Centre, School of Medical Sciences, The University of Sydney, Sydney, NSW 2006, Australia; e Centre for Healthy Ageing, Centenary Institute, Camperdown, NSW 2050, Australia; f School of Sport, Exercise and Rehabilitation Sciences, Faculty of Health, University of Technology Sydney, Sydney, NSW 2007, Australia; g Cellular Bioenergetics Laboratory, Victor Chang Cardiac Research Institute, Darlinghurst, NSW 2010, Australia; h School of Medicine, University of Dundee, Dundee DD1 4HN, UK

**Keywords:** Mitochondrial uncoupling, Diabetes, Obesity, GLP-1, Calorie restriction

## Abstract

Metabolic disorders such as type 2 diabetes, fatty liver disease, hyperlipidemia, and obesity commonly co-occur but clinical treatment options do not effectively target all disorders. Calorie restriction, semaglutide, rosiglitazone, and mitochondrial uncouplers have all demonstrated efficacy against one or more obesity-related metabolic disorders, but it currently remains unclear which therapeutic strategy best targets the combination of hyperglycaemia, liver fat, hypertriglyceridemia, and adiposity. Herein we performed a head-to-head comparison of 5 treatment interventions in the female *db/db* mouse model of severe metabolic disease. Treatments included ~60 % calorie restriction (CR), semaglutide, rosiglitazone, BAM15, and niclosamide ethanolamine (NEN). Results showed that BAM15 and CR improved body weight and liver steatosis to levels superior to semaglutide, NEN, and rosiglitazone, while BAM15, semaglutide, and rosiglitazone improved glucose tolerance better than CR and NEN. BAM15, CR, semaglutide, and rosiglitazone all had efficacy against hypertriglyceridaemia. These data provide a comprehensive head-to-head comparison of several key treatment strategies for metabolic disease and highlight the efficacy of mitochondrial uncoupling to correct multiple facets of the metabolic disease milieu in female *db/db* mice.

## 1. Introduction

Metabolic diseases are conditions associated with abnormal nutrient storage, processing, or utilization. Common examples of metabolic diseases include type 2 diabetes, obesity, fatty liver disease and cardiovascular disorders. The increasing prevalence of metabolic diseases and their complications remains a huge burden globally. Current interventions for metabolic diseases include lifestyle interventions, bariatric surgery, and pharmacotherapies. Lifestyle interventions such as calorie restriction and exercise have poor patient compliance over the long term, and bariatric surgery has risks and costs that limit its adoption on a global scale. In contrast, the development of safe and effective pharmacotherapies represents a potential global solution that could have high compliance.

Patients with metabolic disease frequently take multiple medications for the control of glucose, adiposity, dyslipidaemia, and/or blood pressure [1,2]. Therefore, a pharmacotherapy that targets multiple aspects of metabolic disease would be highly desirable to not only exert beneficial metabolic effects but also to simplify patient management and drug prescription. Several glucose-lowering drugs are currently available on the market, but few glucose-lowering agents target obesity and fatty liver as well as hyperglycaemia. One leading example of a broadly effective metabolic disease therapeutic is semaglutide, a long-acting glucagon-like peptide 1 (GLP-1) analogue that is approved for clinical use [3] and has been shown to both improve weight loss and decrease blood glucose [4]. Semaglutide lowers blood glucose by modulating insulin and glucagon secretion and promotes weight loss by decreasing food intake and gastric motility [5]. However, patients treated with semaglutide frequently experience nausea and adverse gastrointestinal side effects [4].

An alternative approach to decreasing energy intake with semaglutide is to increase energy expenditure through mitochondrial uncoupling [6]. Mitochondrial uncoupling has therapeutic benefits for metabolic disease through dual mechanisms of burning excess nutrients and decreasing mitochondria-derived oxidative stress [6]. In animal models of obesity-related diseases, several mitochondrial uncouplers have shown efficacy to improve glucose tolerance and decrease liver fat, including modified and controlled release versions of 2,4-dinitrophenol [7–9], niclosamide ethanolamine (NEN) [10], and novel molecules such as OPC-163493 [11] and compound 6j [12]. Our group previously demonstrated that the small molecule mitochondrial uncoupler BAM15 increased body fat loss in addition to improving glucose homeostasis, decreasing hypertriglyceridaemia, and decreasing liver steatosis in male C57BL/6 mice fed a 45 % high-fat diet [13] and in male *db/db* mice [14]. However, BAM15 is yet to be tested head-to-head with leading drug interventions used to treat human patients. Furthermore, BAM15 has not yet been tested in female mice in any model of metabolic disease, which is an important gap in the literature. Males and females exhibit differences in physiology, including differences in body composition and nutrient homeostasis [15–17]; therefore, it is important that both sexes are represented in pre-clinical literature.

Herein, we performed a head-to-head comparison of leading pharmacological interventions for metabolic disease alongside calorie restriction and the mitochondrial uncouplers BAM15 and NEN in genetically obese, hepatosteatotic, hypertriglyceridaemic, and hyperglycaemic female *db/db* mice. Semaglutide and the insulin-sensitising thiazolidinedione rosiglitazone are FDA-approved drugs that are currently prescribed for type 2 diabetes, and were selected for comparison because they are among the best-performing molecules in *db/db* mice and humans [4,18,19]. The FDA-approved anti-helminthic drug niclosamide ethanolamine (NEN) was chosen as a benchmark uncoupler compared to BAM15 because it is a molecule that has mitochondrial uncoupling activity as one of its multiple mechanisms of action, and was the only commercially available mitochondrial uncoupler molecule that we could obtain that has reported efficacy in *db/db* mice [10].

The *db/db* mouse model is a rapid and aggressive disease model of metabolic disease that is difficult to overcome [20,21]. We previously compared BAM15 with calorie restriction in male *db/db* mice [14] where the beneficial effects of BAM15 and calorie restriction on glucose control, weight loss, and blood and liver triglyceride levels encouraged us to expand our investigation into female mice and to benchmark against FDA-approved molecules. Research in female mice is important as females are relatively understudied compared to males in the context of metabolic disease and drug intervention which limits the translational potential of outcomes [22]. The results of this study show that all treatment strategies except NEN were effective at reversing at least one metabolic disorder in female *db/db* mice. BAM15 performed better overall as it improved glucose tolerance, decreased liver fat, lowered blood triglyceride, and normalised body weight gain while retaining the largest amount of lean mass as a percent of body mass. BAM15 fully corrected fasting hyperglycaemia and glucose intolerance with efficacy similar to semaglutide and rosiglitazone. However, in contrast to rosiglitazone and semaglutide, BAM15 had better effects to lower body weight. The body weight-lowering effect of BAM15 was comparable to calorie-restricted mice that ate ~60 % fewer calories. Moreover, BAM15 produced greater improvements in liver triglyceride content than both semaglutide and rosiglitazone. Together, our findings highlight the strong effect of the pre-clinical mitochondrial uncoupler BAM15 to target multiple components of the metabolic syndrome.

## 2. Material and methods

### 2.1. Materials

Methylcellulose, Tween80, DMSO, glucose, chloroform, analytical-grade methanol, analytical-grade ethanol, glycerol standard, and formalin were obtained from Sigma-Aldrich. Rosiglitazone and semaglutide were acquired from Cayman Chemical, distributed by Sapphire Bioscience. Niclosamide ethanolamine (NEN) was obtained from Chemlin Chemical Industry. BAM15 was prepared by the Santos Lab at Virginia Tech as previously described [13]. The Insulin ELISA kits were purchased from Crystal Chem. GPO reagent and cholesterol standard were acquired from Pointe Scientific. Infinity cholesterol liquid stable reagent was obtained from ThermoFisher.

### 2.2. Animal husbandry

All mouse experiments were conducted at UNSW and approved by the UNSW Animal Care and Ethics Committee (project approval 20/67A). *Db/db* mice (BKS.Cg-Dock7^m^+/+Lepr^db^/J^Ausb^) were bred at Australian BioResources (Moss Vale, NSW, Australia) from initial breeding pairs provided by the laboratory of Prof Kerry-Anne Rye, UNSW, Australia. *Db/db* mice are a model of severe metabolic disease that replicates many aspects of human obesity and type 2 diabetes [20]. Mice were housed in specific pathogen-free conditions at 22 °C in a light/dark cycle of 12 h and monitored as per ethical guidelines. Unless otherwise stated, mice were provided with *ad libitum* access to water and standard chow diet (Gordons Specialty Feeds, NSW, Australia).

### 2.3. Indirect calorimetry

An Oxymax CLAMS (Columbus Instruments’ Comprehensive Lab Animal Monitoring System, USA) indirect calorimeter was used to analyse energy consumption. 12-week-old *db/db* mice were placed in cages and allowed to acclimatise for at least 24 h while being fed powdered chow food *ad libitum*. Mice were then administered an oral gavage of either vehicle or 100 mg/kg BAM15 in a formulation containing 0.7 % methylcellulose (Sigma M0512, 93 %), Tween80 (Sigma P6474, 2 %) and DMSO (Sigma D5879, 5 %). Oxygen consumption (VO_2_), carbon dioxide output (VCO_2_), and activity based on laser beam breaks were measured. VO_2_ was normalised to lean mass and the respiratory exchange ratio (RER) was calculated as the ratio of VCO_2_ over VO_2_.

### 2.4. Preparation of animal diets

To prepare chow diet containing admixed drug, normal chow food (Gordon’s Specialty Feeds, SF00-100 Irr) was first powdered using a food processor. The same chow diet was used to prepare diet for all treatment groups. Then, powdered diet was mixed with the correct concentration of BAM15 (prepared by Santos Lab at Virginia Tech), niclosamide ethanolamine (NEN, Nanjing Chemlin Chemical Industry Co., Ltd., China), or rosiglitazone (Sapphire Bioscience 21740). Diets were compressed into pellets using a small pellet mill (Gemco ZLSP-120B). For untreated chow diet, powdered diet was pressed into pellets without adding any drug.

### 2.5. Study design

To measure baseline parameters, female *db/*+ and *db/db* mice were assessed for body composition by EchoMRI and glucose tolerance at 5–6 weeks of age, similar to previously published [14]. *Db/db* mice were stratified into treatment groups based on fat mass and glucose tolerance. Mice were housed in groups of 1–3. There were nine treatment groups comprising one *db/*+ group (lean controls) and eight *db/db* groups. Of the eight *db/db* groups, one group was designated the *db/db* control group and fed untreated chow *ad libitum*. Another group was also fed untreated chow *ad libitum* but treated twice weekly with subcutaneous injections of semaglutide (Sapphire Bioscience 29,969) dissolved in 0.5 % DMSO in PBS at a dose of 0.3 mg/kg body weight. Four other groups were fed one of the following diets *ad libitum*: chow containing 0.1 % (w/w) BAM15, 0.2 % (w/w) BAM15, 0.15 % (w/w) NEN, or 0.015 % (w/w) rosiglitazone. The remaining two groups of *db/db* mice were pair-fed. To model calorie restriction (CR), one group was given the same amount of untreated chow each day as the average consumed by the lean *db/*+ mice; this amounted to approximately 60 % calorie restriction. The second pair-fed group consisted of *db/db* mice provided with the same amount of 0.2 % BAM15-containing chow as the average food intake of the *db/db* controls (called the PF 0.2 % BAM15 group). The PF 0.2 % BAM15 group was added for two reasons. First, the calorie-restricted mice inevitably experience time-restricted feeding as they consumed all their food during the first few hours of the dark cycle. Therefore, the PF 0.2 % BAM15 group acted as a control for the time-restricted feeding pattern. Second, pair-feeding diet containing 0.2 % BAM15 to the controls given chow *ad libitum* prevented the possibility of overeating, because it was observed that mice fed 0.2 % BAM15 *ad libitum* tended to eat more; however, this group also tended to shred their food more than other groups such that accurate food intake measurements were difficult. The shredding behaviour was likely due to poor taste of the food resulting from the higher concentration of drug, combined with the less compacted nature of the in-house pellets compared to commercial pellets, owing to the lower pressure achievable using a small pellet mill. All groups had seven mice, except the *db*/+ and *db/db* control groups, which had nine.

Doses of drugs were selected based on previously published data in the literature. NEN at the 0.15 % dose has previously been shown to improve fasting glucose but not body weight in male *db/db* mice [10]. Twice-weekly semaglutide at 0.3 mg/kg has been demonstrated to improve glucose tolerance and decrease body weight in male *db/db* mice [18]. The selected dose of 0.015 % rosiglitazone has been shown to decrease blood glucose, but not body weight, in male and female *db/db* mice [19,23,24]. Following 4 weeks of treatment, body composition was assessed non-invasively using an EchoMRI^™^ Body Composition Analyzer (EchoMRI, Houston, TX, USA) and a glucose tolerance test was performed. At termination, serum was collected by cardiac puncture under isoflurane anaesthesia and separated from whole blood by centrifugation at 2000 ×*g* and 4 °C for 10 min. Mice were euthanised by cervical dislocation and dissected tissues were snap-frozen in liquid nitrogen and stored at −80 °C until analysis.

### 2.6. Glucose tolerance test

Mice were fasted for 6 h during the day prior to the glucose tolerance test (GTT). Mice received an injection of 20 % (w/v) dextrose at a dose of 1 g/kg lean body mass. Blood glucose was measured using an Accuchek Performa glucometer at the time points indicated. When the upper limit of the glucometer was exceeded (blood glucose > 33.3 mM), readings were recorded as 33.3 mM.

### 2.7. Plasma insulin content

Random-fed and fasting blood samples (~40 μL) were collected from the tail tip in heparinised capillary tubes (Sarstedt 16.443). Plasma was extracted after centrifugation at 2000 ×*g* and 4 °C for 10 min. Plasma insulin was measured using a Crystal Chem Ultra-Sensitive Mouse Insulin ELISA Kit (Crystal Chem Inc. 90080, Chicago, IL, USA), according to manufacturers’ instructions except that test samples and standards were incubated overnight at 4 °C. HOMA-IR was calculated as a proxy indicator of insulin resistance as previously described [14].

### 2.8. Serum and liver lipid contents

Frozen liver tissue was powdered in liquid nitrogen using a tissue pulveriser (Cellcrusher, Cork, Ireland). Lipids were extracted using a modified version of the Folch method [25]. In brief, 25 mg of frozen powdered tissue was thoroughly mixed with 533 μL chloroform and 267 μL methanol. Samples were then sonicated for 10 min and digested at room temperature for 45 min. Then, 400 μL of 0.9 % sodium chloride was added and samples were centrifuged at 600 ×*g* at room temperature for 10 min. The bottom layer was extracted and dried under nitrogen gas in a TurboVap^®^ Evaporator (Biotage, Uppsala, Sweden). Lipids were dissolved in 0.4 mL 95 % (v/v) ethanol and heated to 37 °C prior to lipid assays. Triglyceride and cholesterol levels were measured in lipid extracts from frozen liver and undiluted serum. Triglyceride levels were measured using GPO reagent (Pointe Scientific T7532, Canton, MI, USA) according to the manufacturer’s protocol, with glycerol standard (Sigma G7793, St Louis, MO, USA). Cholesterol levels were measured using Infinity cholesterol liquid stable reagent (ThermoFisher TR13421) according to the manufacturer’s protocol, using cholesterol standard (Pointe Scientific C7509, Canton, MI, USA).

### 2.9. Liver histology

At termination, the left lobe of the liver was fixed in 10 % formalin at room temperature overnight, then transferred to 70 % ethanol and stored at 4 °C until processing. Sample processing and staining was performed by the Biological Specimen Preparation facility at the Mark Wainwright Analytical Centre, UNSW Sydney. Samples were embedded in paraffin and 4 μm sections were cut using a microtome. Immediately prior to staining, slides were dewaxed by baking for 60 min at 58 °C then put through a xylene/ethanol series. Haematoxylin-eosin (H&E) staining was performed, and slides were dehydrated and mounted.

### 2.10. Data and statistical analysis

All data points were collected from discrete biological replicates and are presented as the mean ± SEM. For glucose tolerance testing, data points were excluded from one mouse in the NEN group at baseline that did not receive a successful i.p. injection of glucose. For quadriceps weight, results were inadvertently not collected in 2 *db*/+, 2 *db/db* control and 1 *db/db* 0.2 % BAM15 mice. Sample size was determined using G*Power (v.3.1.9.7) with 80 % power and 5 % alpha with GTT-AUC as the primary outcome.

Statistical tests were conducted to determine differences between treatment groups. To compare more than two groups, One-Way ANOVA was performed with Tukey’s multiple comparisons test to compare each group to every other group. For clarity, only significant differences compared to *db/db* control are illustrated on the figures; other significant changes are described in the text. Indirect calorimetry data were analysed by Two-Way Repeated Measures ANOVA with Dunnett’s multiple comparisons test. The threshold for statistical significance was *p* < 0.05 using Prism (v.9.4.1; GraphPad Software).

## 3. Results

### 3.1. BAM15 bioactivity in db/db mice

**Fig. 1.**
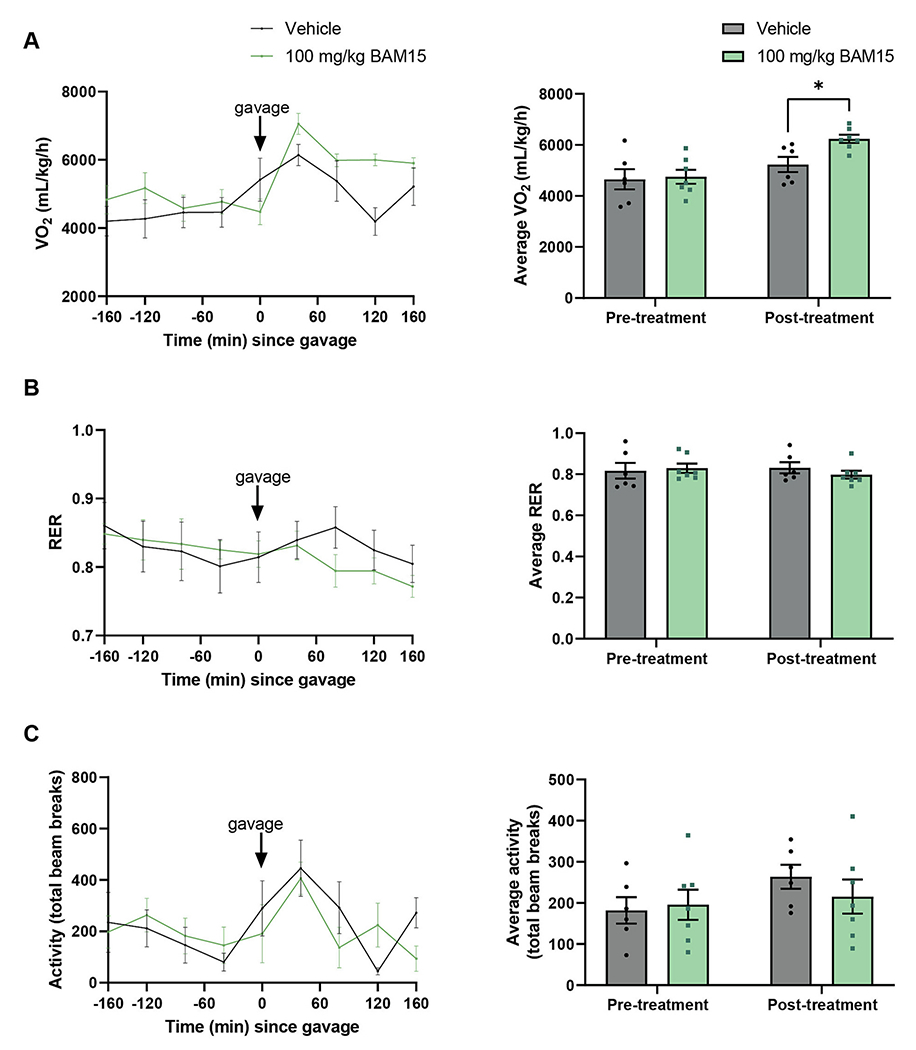
BAM15 increased oxygen consumption in female *db/db* mice. Oxygen consumption normalised to lean body mass (A), respiratory exchange ratio (RER, B) and locomotor activity as number of laser beam breaks (C). Pre-treatment refers to the 160 min before oral gavage and post-treatment refers to the 160 min following oral bolus dose of vehicle or BAM15. * indicates *p* < 0.05 as assessed by Two-Way ANOVA. Graphs show mean ± SEM. *n* = 6–7 per group.

Mitochondrial uncouplers increase energy expenditure by definition of their mechanism of action. We have recently shown that BAM15 increases energy expenditure in male *db/db* mice [14], and the bioactivity of BAM15 was also investigated in female *db/db* mice herein for purposes of due diligence. Female *db/db* mice were subjected to indirect calorimetry over a period of ~3 h before and after treatment with an oral gavage of BAM15 at 100 mg/kg body weight. BAM15 was administered as an acute oral bolus rather than admixed in food, as whole-body changes in oxygen consumption are subtle and subject to variability when assessing unrestrained free-moving mice that consume BAM15 in diet at random. The 100 mg/kg dose is the same as tested in male *db/db* mice [14], and we observed a similar phenotype in females whereby BAM15 treatment significantly increased oxygen consumption rate (*V*O_2_) by ~19 % compared to vehicle-treated controls over the first 160 min of treatment (Fig. 1A) without a significant change in respiratory exchange ratio (RER) or locomotor activity (Figs. 1B,C). The lack of change in locomotor activity demonstrates that increased VO_2_ was not due to hyperactivity. The acute 100 mg/kg dose is approximately one-fifth of the estimated dose of BAM15 consumed per day in female *db/db* mice given BAM15 admixed in food at 0.2 % (w/w) (*e.g.,* 8 g food consumption for a 30-gram mouse).

### 3.2. Body weight and body composition

**Fig. 2.**
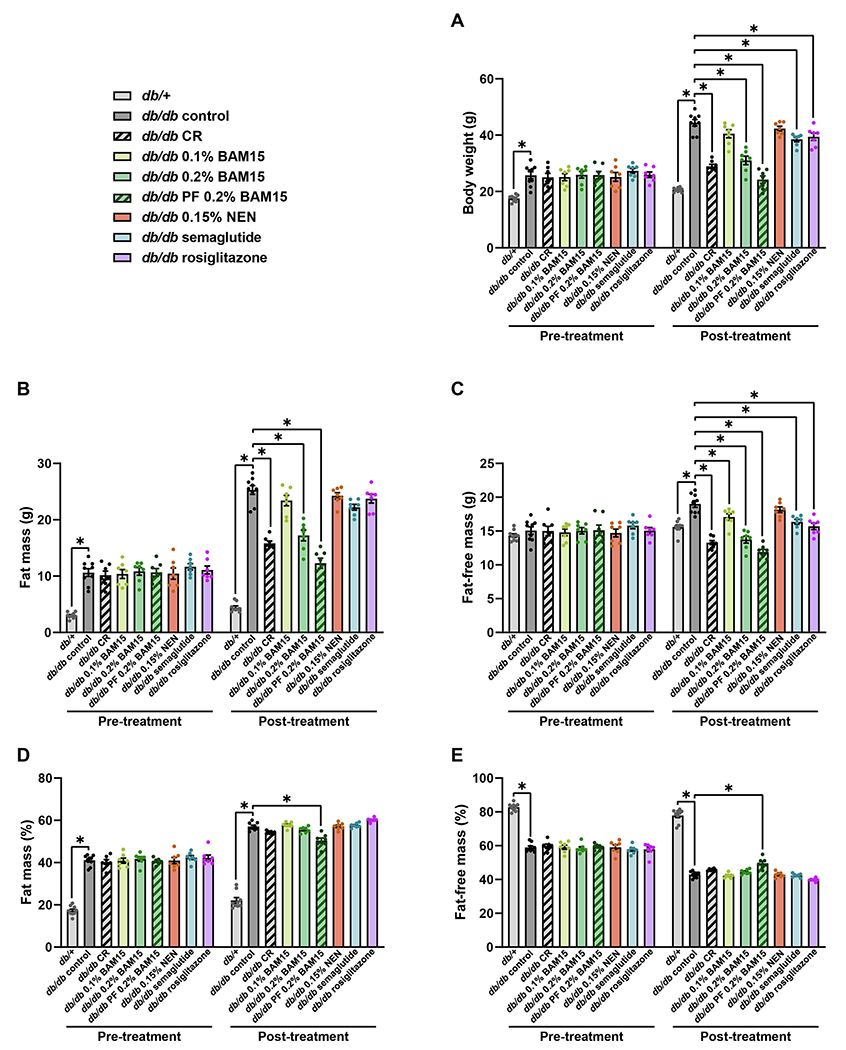
Body weight and composition of female *db*/+ and *db/db* mice after 4 weeks of treatment. Body weight (A), fat mass (B), fat-free lean mass (C), percentage fat mass (D), and percentage fat-free lean mass (E). Statistical significance was analysed by One-Way ANOVA with Tukey’s multiple comparisons test, comparing each group to every other group. Only significant changes (p < 0.05) compared to *db/db* control are shown, indicated by *. Graphs show mean ± SEM. *n* = 7–9 mice per group.

To investigate the effects of pharmacologic and diet interventions on body weight and body composition, 8 groups of female *db/db* mice and one group of female *db*/+ mice were treated for 4 weeks. At the end of the study period, *db/db* control mice fed normal chow diet *ad libitum* had approximately twice the body weight of *db/*+ mice (Fig. 2A). Both calorie restriction (CR) and 0.2 % BAM15 treatments markedly decreased body weight by ~30 % (Fig. 2A), while semaglutide and rosiglitazone produced moderate decreases in body weight of ~12 % (Fig. 2A). The body weights of the CR and both 0.2 % BAM15 groups (*ad libitum* or pair-fed (PF) to *db/db* controls) were significantly lower than those of the semaglutide and rosiglitazone groups (*p* = 0.0004 for 0.2 % BAM15 *vs* semaglutide, *p* < 0.0001 for others). In contrast, neither the low dose of 0.1 % BAM15 nor 0.15 % NEN significantly altered body weight (Fig. 2A). EchoMRI was performed to estimate the fat and fat-free mass in the animals and revealed that the changes in body weight in the CR, 0.2 % BAM15 *ad libitum*, semaglutide, and rosiglitazone groups occurred as proportional losses of fat mass and fat-free mass (Fig. 2B–E). Both fat mass and fat-free mass were significantly decreased by CR and 0.2 % BAM15 *ad libitum* compared to *db/db* controls (Fig. 2B–C), but animals maintained normal body composition as the percentages of fat mass and fat-free mass over total body mass were not altered (Fig. 2D–E). Similarly, among the *db/db* mice given 0.1 % BAM15, semaglutide, and rosiglitazone, the proportions of fat mass and fat-free mass were unchanged compared to *db/db* controls (Fig. 2D–E), even though fat-free mass, but not fat mass, was slightly and significantly decreased (Fig. 2B–C). In contrast, the PF 0.2 % BAM15 group lost more fat mass than fat-free mass, resulting in a decreased percentage fat mass (Fig. 2D) and increased percentage fat-free mass (Fig. 2E) compared to *db/db* controls. Among drug-treated mice, body weight losses were not due to decreased food intake, as the 0.1 % BAM15, semaglutide and rosiglitazone groups consumed the same amount of food as *db/db* control mice (Fig. S1). Food intake was more difficult to measure in mice fed 0.2 % BAM15 as they tended to shred the food to a greater extent than other groups, which made it difficult to accurately measure food intake. Therefore, these mice appeared to eat more than *db/db* controls (Fig. S1), and the pair-fed 0.2 % BAM15 group was included to overcome this potential confounder.

**Fig. 3.**
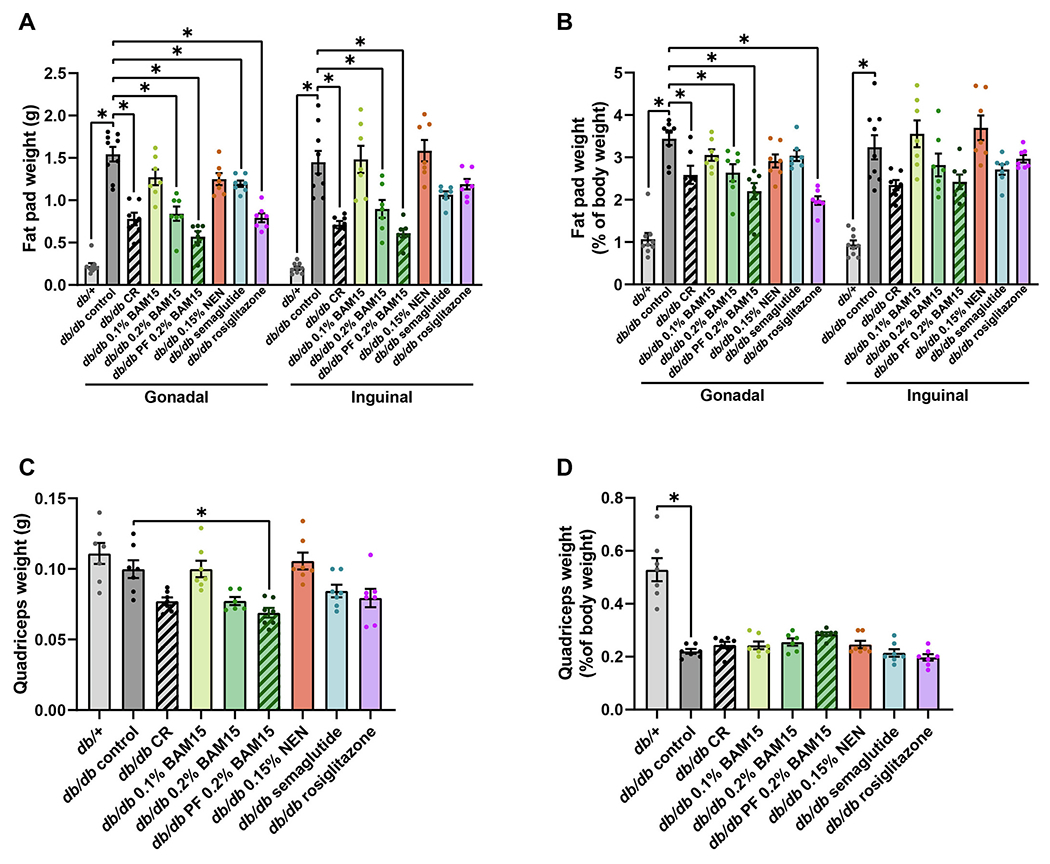
Tissue wet weights in female *db*/+ and *db/db* mice after 4 weeks of treatment. Weights of single gonadal and inguinal fat pads (A) and relative fat pad weight normalised to body weight (B). Quadriceps mass as absolute weights (C) and normalised to body weight (D). Statistical significance was analysed by One-Way ANOVA with Tukey’s multiple comparisons test, comparing each group to every other group. Only significant changes (p < 0.05) compared to *db/db* control are shown, indicated by *. Graphs show mean ± SEM. n = 6–9 mice per group.

Tissue wet weights were measured at termination to assess changes more directly in the masses of fat pads and muscle. The average weight of the gonadal fat pad, which represents visceral fat, was ~7 times higher in *db/db* controls than in *db/*+ mice (Fig. 3A). Gonadal fat pad weight was approximately halved by CR, both 0.2 % BAM15 treatments, and rosiglitazone, decreased by ~20 % by semaglutide, and moderately but non-significantly decreased by 0.1 % BAM15 and NEN (Fig. 3A). As the mice from different treatment groups differed in body weight, fat pad weights were also normalised to body weight. As a percentage of body weight, gonadal fat pad weight in *db/db* mice remained lower in all treatment groups than *db/db* controls, which was statistically significant in the CR, both 0.2 % BAM15, and rosiglitazone groups (Fig. 3B).

Similar to the gonadal fat, the subcutaneous inguinal fat depot was ~7 times higher in *db/db* control mice compared to lean *db*/+ mice (Fig. 3A). Inguinal fat pad weight was significantly decreased by ~40 % in the CR, 0.2 % BAM15 and PF 0.2 % BAM15 groups (Fig. 3A). Semaglutide and rosiglitazone produced moderate decreases in inguinal fat pad weight compared to *db/db* controls, but these changes were not significant (Fig. 3A). Treatment with 0.1 % BAM15 and NEN did not alter inguinal fat pad weight (Fig. 3A). When normalised to body weight, inguinal fat weight was greater in *db/db* controls than *db*/+ mice, and there was a non-significant trend towards decreased inguinal fat weight as a percentage of body weight in the CR, 0.2 % BAM15, PF 0.2 % BAM15, semaglutide and rosiglitazone groups (Fig. 3B).

Overall, there were minimal changes in muscle mass as a result of the drug and calorie restriction treatments. Absolute quadriceps weight was decreased by pair-feeding 0.2 % BAM15, and a non-significant trend for decreased quadriceps mass was observed with CR, 0.2 % BAM15 *ad libitum*, semaglutide, and rosiglitazone treatments (Fig. 3C). However, when quadriceps weight was normalised as a percentage of total body weight, there were no differences among the *db/db* groups with the pair-fed 0.2 % BAM15 group trending to have the greatest quadriceps mass of all *db/db* mice when normalised to body mass (Fig. 3D).

### 3.3. Treatment effects on glucose tolerance

**Fig. 4.**
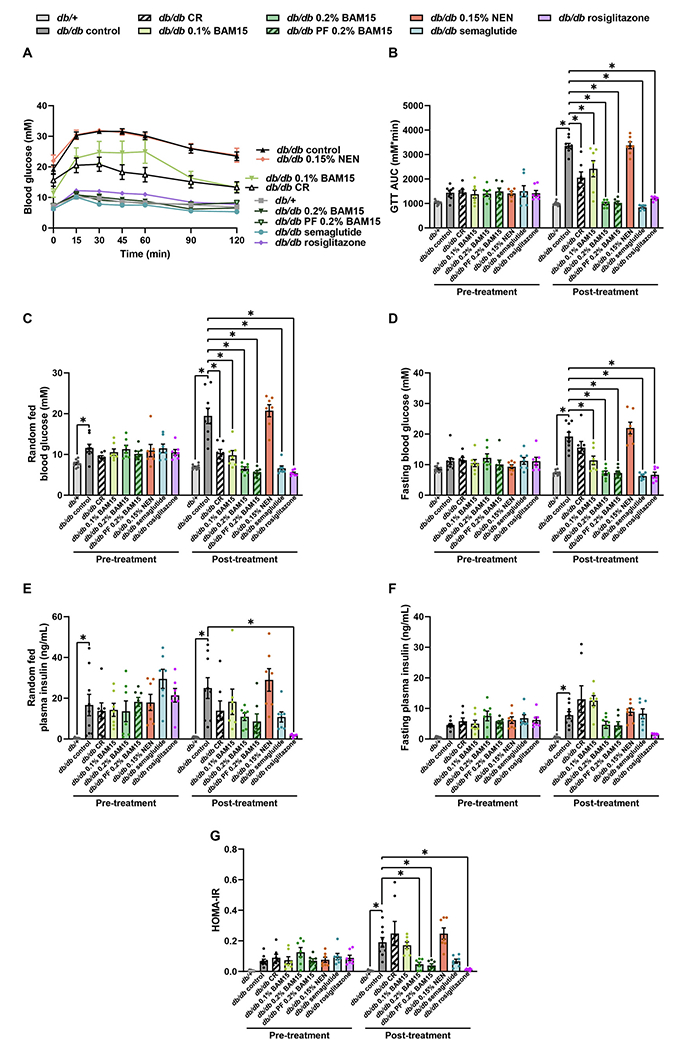
Glucose tolerance, blood glucose and plasma insulin levels in female *db*/+ and *db/db* mice after 4 weeks of treatment. Glucose tolerance curve (A) and area under the curve (AUC) (B). Random-fed (C) and fasting (D) blood glucose concentrations. Random-fed (E) and fasting (F) plasma insulin concentrations. Calculated HOMA-IR (G). Statistical significance was analysed by One-Way ANOVA with Tukey’s multiple comparisons test, comparing each group to every other group. Only significant changes (p < 0.05) compared to *db/db* control are shown, indicated by *. Graphs show mean ± SEM. n = 7–9 mice per group.

Glucose tolerance tests (GTTs) were conducted to determine the effects of CR and drug treatments on blood glucose control. After 4 weeks of treatment, the *db/db* control mice and NEN-treated mice showed the most severe glucose intolerance; these groups were not different from each other and showed very high fed and fasting glucose levels (~20 mM) and exceeded the limit of the glucometer (33.3 mM) at many time points during the GTT. When the glucometer reported a reading of ‘HI’, we used a value of 33.3 mM for data calculations; therefore, glucose intolerance in the *db/db* control mice and NEN-treated mice is underestimated (Fig. 4A–B). Partial improvement in glucose tolerance was observed in the 0.1 % BAM15 and CR treatment, which is evidenced by decreased AUC of the GTT curves and fed blood glucose levels (Fig 4A–C). Fasting blood glucose was also significantly improved by 0.1 % BAM15 (Fig. 4D).

The treatments that had the best effect on glucose tolerance were 0.2 % BAM15, semaglutide, and rosiglitazone, which all completely restored glucose tolerance, fed blood glucose, and fasting blood glucose to levels comparable to lean *db*/+ controls (Fig. 4A–D). The area under the curve for the glucose tolerance test was significantly lower in both 0.2 % BAM15 groups, the semaglutide group, and the rosiglitazone group, compared to each of the CR and 0.1 % BAM15 groups (all relevant comparisons *p* ≤ 0.0037). Similarly, fasting glucose remained higher in the CR group, but not the 0.1 % BAM15 group, compared to both 0.2 % BAM15 groups, the semaglutide group, and the rosiglitazone group (*p* ≤ 0.0005 for CR *vs* each of these listed groups). Treatment with 0.15 % NEN did not change glucose tolerance or fed and fasting blood glucose concentrations (Fig. 4B–E).

At the end of the study period, *db/db* controls were hyper-insulinaemic compared to *db*/+ mice, with 32-fold higher random-fed insulin and 14-fold higher fasting insulin (Fig. 4E–F). In the random-fed state, plasma insulin levels were significantly decreased 93 % in the rosiglitazone group compared to *db/db* controls (Fig. 4E). The mice treated with CR, 0.1 % BAM15, 0.2 % BAM15 *ad libitum*, 0.2 % BAM15 pair-feeding, and semaglutide showed trends towards decreased random-fed insulin levels (Fig. 4E). In the fasted state, no treatment significantly decreased plasma insulin levels compared to *db/db* controls, but mice treated with CR and 0.1 % BAM15 showed trends towards increased plasma insulin, while mice given 0.2 % BAM15 and rosiglitazone showed trends towards decreased plasma insulin (Fig. 4F). NEN did not change random-fed or fasting insulin levels (Fig. 4E–F). HOMA-IR was used as an indicator of insulin resistance (Fig 4G). HOMA-IR was increased in *db/db* controls compared to *db*/+ mice, and significantly decreased by both 0.2 % BAM15 treatments and rosiglitazone (Fig. 4G). A trend for decreased HOMA-IR was observed in the semaglutide group (Fig 4G). The CR group had a trend for increased HOMA-IR compared to *db/db* controls and had significantly higher HOMA-IR than both 0.2 % BAM15 groups, the semaglutide group, and the rosiglitazone group (*p* ≤ 0.0067 comparing CR to each listed group).

### 3.4. Treatment effects on hepatic and serum triglyceride levels

**Fig. 5.**
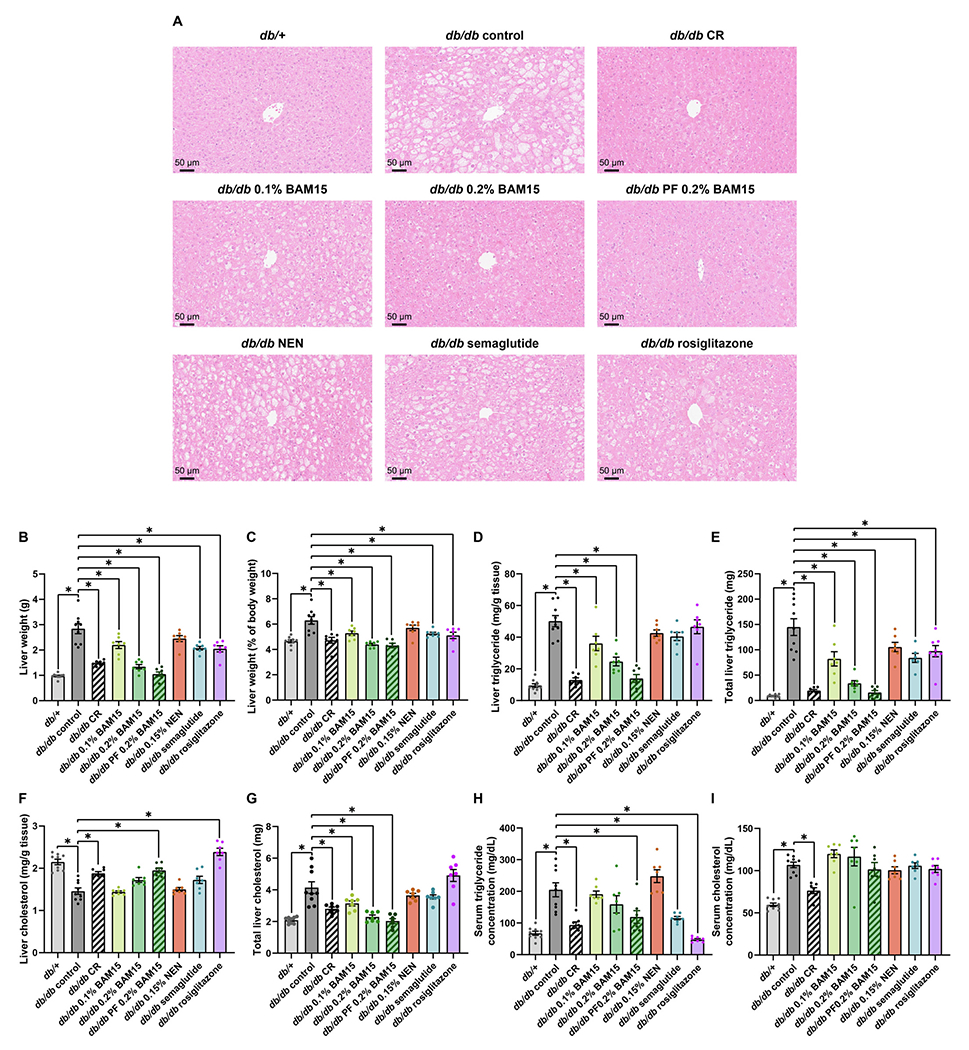
Liver and serum lipid contents in female *db*/+ and *db/db* mice after 4 weeks of treatment. Representative images of liver sections stained with haematoxylin and eosin (A). Liver wet weight in grams (B) and normalised over total body weight (C). Liver triglyceride content per gram of tissue (D) and total triglyceride in liver (E). Liver cholesterol content per gram of tissue (F) and total cholesterol in liver (G). Serum triglyceride (H) and cholesterol (I) concentrations. Statistical significance was analysed by One-Way ANOVA with Tukey’s multiple comparisons test, comparing each group to every other group. Only significant changes (p < 0.05) compared to *db/db* control are shown, indicated by *. Graphs show mean ± SEM. n = 7–9 mice per group.

Liver steatosis is a hallmark feature of metabolic disease that is present in *db/db* mice [26]. Therefore, we investigated the impact of the treatment interventions on fatty liver parameters. Liver tissues were sectioned and stained with haematoxylin and eosin (H&E) (Fig. 5A). Liver wet weight was almost 3 times higher in *db/db* controls compared to lean *db*/+ mice, but *db/db* liver weight was significantly decreased by 48 % by CR, 53 % by 0.2 % BAM15, and 63 % by PF 0.2 % BAM15 (Fig. 5B). The lower dose of 0.1 % BAM15, semaglutide, and rosiglitazone resulted in more moderate but significant decreases in liver wet weight compared to *db/db* controls, while NEN resulted in a mild but non-significant decrease in liver wet weight (Fig. 5B). The same trends are reflected when the liver weight is expressed as a proportion of total body weight (Fig. 5C).

Liver triglyceride levels were measured in frozen liver tissue and found to be 5 times higher in *db/db* controls compared to lean *db* + mice (50 ± 2.5 *vs* 9.5 ± 1.8 mg/g) (Fig. 5D), but were markedly reduced >3-fold by CR (13 ± 1.3 mg/g) and PF 0.2 % BAM15 (14 ± 2.5 mg/g). 0.1 % BAM15 and 0.2 % BAM15 treatments also showed significant and dose-dependent effects to decrease liver triglyceride levels (36 ± 4.5 and 25 ± 2.8 mg/g, respectively) compared to *db/db* controls (Fig. 5D). NEN, semaglutide and rosiglitazone mildly but non-significantly decreased liver triglyceride content relative to grams of liver tissue (Fig: 5D). Liver triglyceride contents in the mice given NEN, semaglutide, and rosiglitazone were significantly higher compared to those in mice in the CR and both 0.2 % BAM15 groups (all relevant comparisons *p* < 0.0137). When total liver triglyceride content was estimated by multiplying the concentration per gram of tissue and the liver wet weight, all treatments except for NEN decreased total liver triglyceride compared to *db/db* controls (Fig. 5E).

Liver cholesterol content was also measured in frozen liver tissue. When presented as liver cholesterol per gram of liver tissue, cholesterol levels appeared to be decreased in *db/db* controls compared to *db*/+ mice, and were increased by CR, pair-feeding 0.2 % BAM15, and rosiglitazone (Fig. 5F). This is likely due to the larger size of the *db/db* control livers, such that cholesterol appeared reduced when normalised over liver tissue weight. Therefore, the total liver cholesterol was estimated by multiplying the cholesterol per gram of tissue with the liver wet weight (Fig. 5G). Total liver cholesterol content was approximately twice as high in *db/db* controls as *db*/+ mice but was significantly decreased by 0.1 % BAM15, both modes of 0.2 % BAM15 administration, and CR (Fig 5G). Total liver cholesterol was not changed by 0.15 % NEN, semaglutide, or rosiglitazone compared to *db/db* controls; these groups also had higher total liver cholesterol compared to both 0.2 % BAM15 groups (all relevant comparison *p* ≤ 0.0060).

Serum triglyceride levels were ~ 3-fold greater in *db/db* control mice than *db*/+ controls (Fig. 5H). Treatment interventions that significantly decreased serum triglyceride levels included a 54 % decrease in the CR group, 43 % decrease in the PF 0.2 % BAM15 group, 44 % decrease in the semaglutide group, and 77 % in the rosiglitazone group (Fig. 5H). Serum cholesterol content was approximately 1.8-fold greater in *db/db* control mice than *db*/+ controls, with only CR significantly decreasing serum cholesterol levels by 29 % (Fig. 5I).

**Fig. 6.**
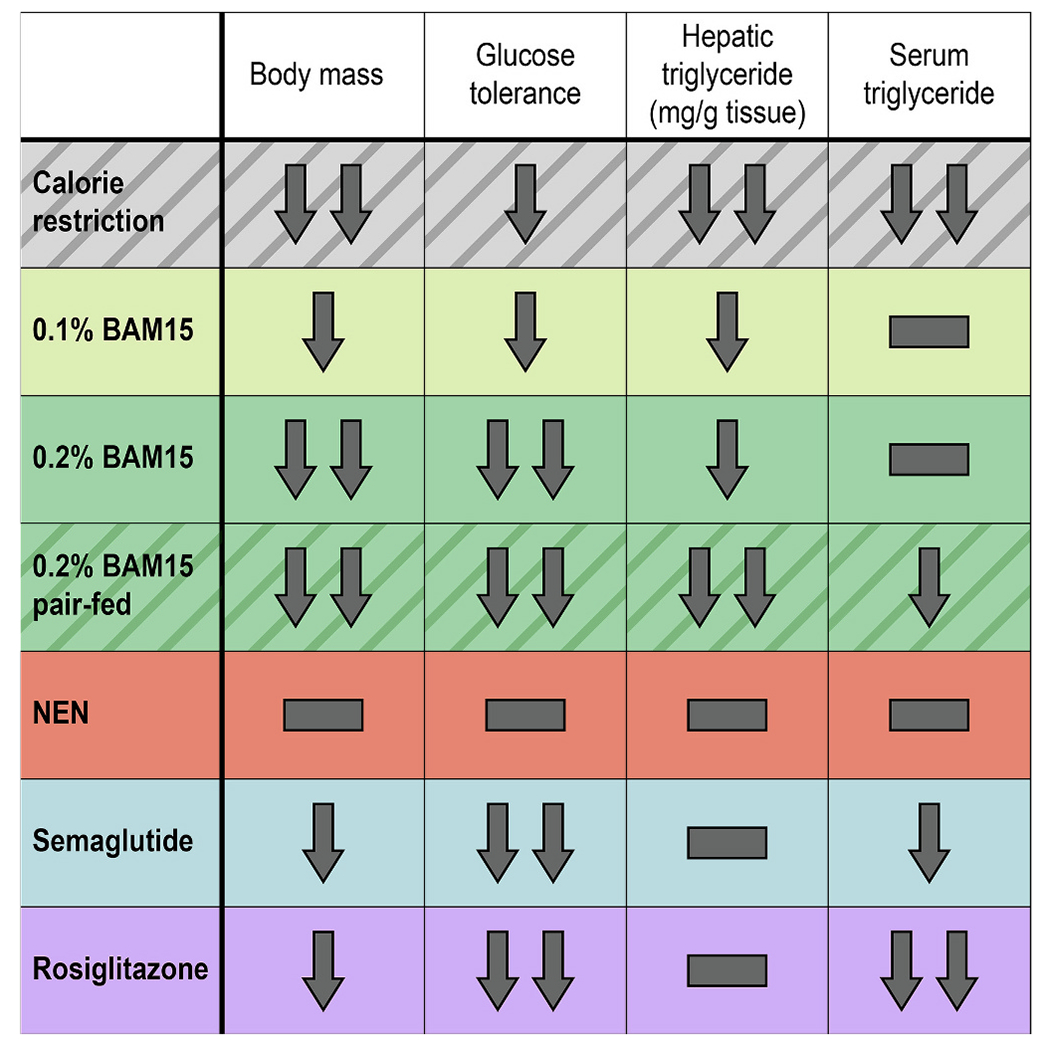
Summary of major effects of drug treatment and calorie restriction in female *db/db* mice. One arrow = moderate decrease, two arrows = marked decrease, hyphen = no change.

Overall, calorie restriction and 0.2 % BAM15 caused the greatest decreases in body mass, and 0.2 % BAM15, semaglutide, and rosiglitazone had the strongest effects on blood glucose homeostasis. Calorie restriction and the pair-fed 0.2 % BAM15 treatment resulted in the greatest anti-hepatosteatotic effects, while the strongest decreases in serum triglyceride content were achieved by calorie restriction and rosiglitazone (Fig. 6).

## 4. Discussion

In this study, we performed a head-to-head comparison of five treatment interventions that have shown strong efficacy to reverse metabolic disease phenotypes in either pre-clinical or clinical studies using the female *db/db* mouse model. The interventions included 60 % calorie restriction, the GLP-1 analogue semaglutide, the insulin sensitiser rosiglitazone, and two mitochondrial uncouplers including BAM15 and NEN. Our results demonstrate that BAM15 exerted beneficial effects on the most metabolic disease parameters, with weight loss and anti-hepatosteatotic effects that were comparable to calorie restriction, and glucose-lowering effects that were comparable to anti-diabetes drugs semaglutide and rosiglitazone.

BAM15 treatment resulted in dose-dependent effects on body weight, glucose tolerance, and liver triglyceride content. These results were remarkably reproducible and consistent with previous results in male *db/db* mice, in which BAM15 treatments had a similar magnitude of effect [14]. For instance, in both the present study in females and our previous study in males, the low dose of 0.1 % BAM15 resulted in improvements in glucose tolerance and insulin levels that were strikingly similar to those of calorie restriction, as both treatments partially improved glucose tolerance and showed a trend for increased plasma insulin levels. Therefore, it is possible that the lower dose of 0.1 % BAM15 is sufficient to mimic the effects of CR on the liver and endocrine pancreas, despite not causing weight loss. In contrast, the higher dose of 0.2 % BAM15 caused a sufficient calorie deficit to achieve a similar level of weight loss as CR, while stimulating strong insulin-sensitising effects to completely restore glucose tolerance to levels similar to *db*/+ mice, an effect that was consistent with previous results in males [14]. The striking consistency between this present study and previous work in male *db/db* mice [14] suggests that BAM15 and calorie restriction interventions act through mechanisms that are not noticeably impacted by any potential influences from sex hormones or sex-dependent gene expression. However, there was a subtle difference in food intake among *db*/+ controls; in our hands, female *db*/+ mice ate slightly less than male *db*/+ mice (~3 g/day *vs* ~4 g/day), but female *db/db* mice were similarly hyperphagic as their male counterparts (~8 g/day). As a result, the calorie restriction group in our previous study in male *db/db* mice amounted to ~50 % calorie restriction when pair-fed to *db*/+ controls [14], while the equivalent group in female *db/db* mice were ~ 60 % calorie-restricted.

The higher dose of 0.2 % BAM15 and calorie restriction treatments were the two interventions that resulted in the greatest body weight change compared to *db/db* control mice. Both 0.2 % BAM15 and calorie restriction interventions had less fat mass and fat-free mass compared to *db/db* controls, while semaglutide and rosiglitazone had less fat-free lean mass but not fat mass. All *db/db* treatment groups except the pair-fed 0.2 % BAM15 group maintained a body composition similar to *db/db* mice, demonstrating that the weight difference did not affect normal body composition proportions of fat and lean mass for *db/db* mice (approx 60 % fat and 40 % lean). In contrast, the *db/db* mice pair-fed 0.2 % BAM15 had lower body fat with greater lean mass as a percentage of body weight. These data on body composition should be interpreted in the context of the *db/db* model, as these mice lack functional leptin receptor and cannot effectively monitor or regulate their body fat stores. *Db/db* mice are known to maintain a very high percentage of body fat compared to lean controls [20]. *Db/db* mice can defend their body fat even after serious interventions, such as bariatric surgery, where they still maintain approximately 60 % fat and 40 % lean mass [27]. However, in diet-induced obese wild-type C57BL/6 mice, we have previously shown that BAM15 decreases fat mass but not fat-free lean mass [13], and others have shown a similar result for semaglutide [28]. Nevertheless, with these caveats in mind, the data in this study show that female *db/db* mice pair-fed BAM15 had the highest ratio of lean mass as a percent of body composition. These data are potentially important because the preservation of lean mass is highly desired in weight loss treatment [29].

Semaglutide was chosen for this study as it is a glucagon-like peptide 1 (GLP-1) analogue that is currently approved for blood glucose management in type 2 diabetes [3] and chronic weight management for overweight and obesity [30]. Semaglutide has been approved for glucose-lowering since 2017 [3] and obesity since 2021 [30] and has been so widely prescribed since its market launch that there have been worldwide shortages [31,32]. GLP-1 analogues work by mimicking the native incretin GLP-1, enhancing insulin secretion, promoting satiety, and improving liver function, together causing weight loss and improved blood glucose control [33,34]. In the current study, semaglutide had excellent effects on blood glucose in *db/db* mice by effectively normalising glucose tolerance to levels comparable with lean healthy controls, and 0.2 % BAM15 was able to match these effects while decreasing body weight and liver triglyceride content to a greater extent.

Rosiglitazone was chosen for a benchmarking comparison in this study as it acts through a different mechanism of action to both semaglutide and BAM15, and has strong glucose-lowering effects in *db/db* mice [19,23]. Rosiglitazone is a thiazolidinedione that increases insulin sensitivity by activating peroxisome proliferator-activated receptor gamma (PPARγ) [35]. Although rosiglitazone is approved as a second-line medication for glucose management in diabetes, its use in the clinic has been limited due to the side effect of weight gain [36–38] and a perceived increased risk of cardiovascular events, which was later refuted [39]. However, rosiglitazone has excellent effects on glucose control in *db/db* mice. In the current study, rosiglitazone improved glucose tolerance to a similar extent as semaglutide and 0.2 % BAM15 and resulted in the greatest decrease in circulating insulin and HOMA-IR among all treatment groups. Rosiglitazone also decreased serum triglyceride levels to the greatest extent. However, similar to semaglutide, rosiglitazone did not decrease body weight or liver triglyceride levels to the same degree as 0.2 % BAM15. We also observed a side effect of considerable fat deposition in the interscapular region of all rosiglitazone-treated mice that was palpable as a hard mass beneath the skin. Upon termination, dissection revealed that this mass consisted of white adipose tissue. Although this did not appear to affect the condition of the animals, it was nevertheless an unwanted side effect. In future studies, one avenue for investigation could involve exploring a combination therapy regime that supplements rosiglitazone with BAM15, to take advantage of the fat-reducing effects of BAM15 while capitalising upon the insulin-sensitising effects of rosiglitazone.

The multiple beneficial effects of BAM15 are attributable to its mechanism of action as a mitochondrial uncoupler, which was confirmed by the indirect calorimetry data showing increased energy expenditure. Mitochondrial uncoupling can simultaneously improve multiple facets of metabolic disease by increasing nutrient oxidation, which addresses the fundamental problem of calorie excess that underlies many obesity-associated conditions. Increased nutrient oxidation is achieved by transporting protons from the intermembrane space into the mitochondrial matrix independent of ATP synthase, resulting in greater nutrient oxidation to support the same level of ATP production. Mitochondrial uncoupling also decreases the rate of production of reactive oxygen species (ROS), which is correlated with the strength of the proton gradient. A previous study on BAM15 in high-fat diet-fed mice suggested that BAM15 administration may increase AMPK activation in the liver to modulate hepatic glucose handling and insulin sensitivity [40]. Our previous work in male *db/db* mice also showed that BAM15 lowered serum glucagon concentration and decreased liver expression of gluconeogenic enzymes [14], suggesting that decreased hepatic glucose production may be an important factor that contributes to the improved blood glucose in BAM15-treated mice. However, the diverse effects of BAM15 on the liver and other tissues remain to be fully elucidated.

Niclosamide is an anti-helminthic drug that also has uncoupling properties [41]. In its ethanolamine salt form, niclosamide ethanolamine (NEN) has been shown to increase oxygen consumption in NIH-3 T3 fibroblasts even in the presence of the ATP synthase inhibitor oligomycin, and increased energy expenditure in high-fat diet-fed mice [10]. However, despite being previously published to improve blood glucose in *db/db* mice [10], 0.15 % NEN in the current study did not affect blood glucose control or any of the parameters measured apart from a slight trend for mildly decreased gonadal fat pad mass and liver triglyceride content. The lack of efficacy of NEN observed in this study is consistent with other studies in male *db/db* mice by us [14] and Hinder et al. [42], which also showed little effect of 0.15 % NEN admixed in chow diet.

A major strength of this study is that it makes progress towards bridging a gap in the literature by using female *db/db* mice, which allows comparison with prior work in males [14]. Pre-clinical studies that include or acknowledge both sexes are important for maximising their translational relevance. The *db/db* mouse model was chosen for this study as it mimics several aspects of human metabolic disease, including obesity, insulin resistance, hyperglycaemia, and fatty liver, and presents a severe phenotype that is difficult to reverse. However, the *db/db* model is not without limitations; for example, as mentioned above, *db/db* mice are leptin-deficient and have low lean mass as a percentage of total body mass. Future work in non-genetic models (*e.g.*, diet-induced obese mice) would be valuable for comparing the effects of BAM15 and calorie restriction on body composition and appetite. Moreover, while the number of replicates used in our study (*n* = 7–9 mice/group) provide sufficient power for resolving many differences, future studies employing larger group sizes may yield greater insight into subtle differences between multiple treatment groups. Another direction for future research would be to investigate the molecular mechanisms that underly the effects of BAM15 and distinguish them from those of semaglutide, rosiglitazone, and calorie restriction. For example, there may be changes in hepatic and peripheral nutrient metabolism that influence insulin sensitivity, glucose clearance, and lipid metabolism, which would explain the phenotypes observed in this study. Finally, an important limitation is that these studies were conducted entirely in female *db/db* mice. While the results provide important clues with potential clinical implications, the effects of BAM15, semaglutide, rosiglitazone, NEN, and calorie restriction in *db/db* mice cannot be assumed to be directly indicative of those that would occur in humans.

Overall, these data demonstrate the strengths and weaknesses of different drugs for improving metabolic disease phenotypes in female *db/db* mice. High-dose BAM15 and calorie restriction were most effective for weight loss and decreasing liver steatosis, while high-dose BAM15, semaglutide, and rosiglitazone had the best effects on glucose tolerance. Low-dose BAM15 was also efficacious, and improved glucose homeostasis independent of changes in body weight or body composition. BAM15’s beneficial effects on multiple metabolic phenotypes suggest that mitochondrial uncouplers may represent a drug class that can be developed to simultaneously treat many metabolic defects common in the metabolic syndrome.

## Supplementary Material

supplemental fig s1

## Data Availability

Data will be made available on request.
