# Supplementary material for: Head-to-head comparison of BAM15, semaglutide, rosiglitazone, NEN, and calorie restriction on metabolic physiology in female db/db mice
Source: Biochim Biophys Acta Mol Basis Dis. Author manuscript; Available in PMC 2024 Sep 1. (PMC10908303; doi:10.1016/j.bbadis.2023.166908)
Supplement: supplemental fig s1 [file NIHMS1969904-supplement-supplemental_fig_s1.docx]

**Supplementary Figure**


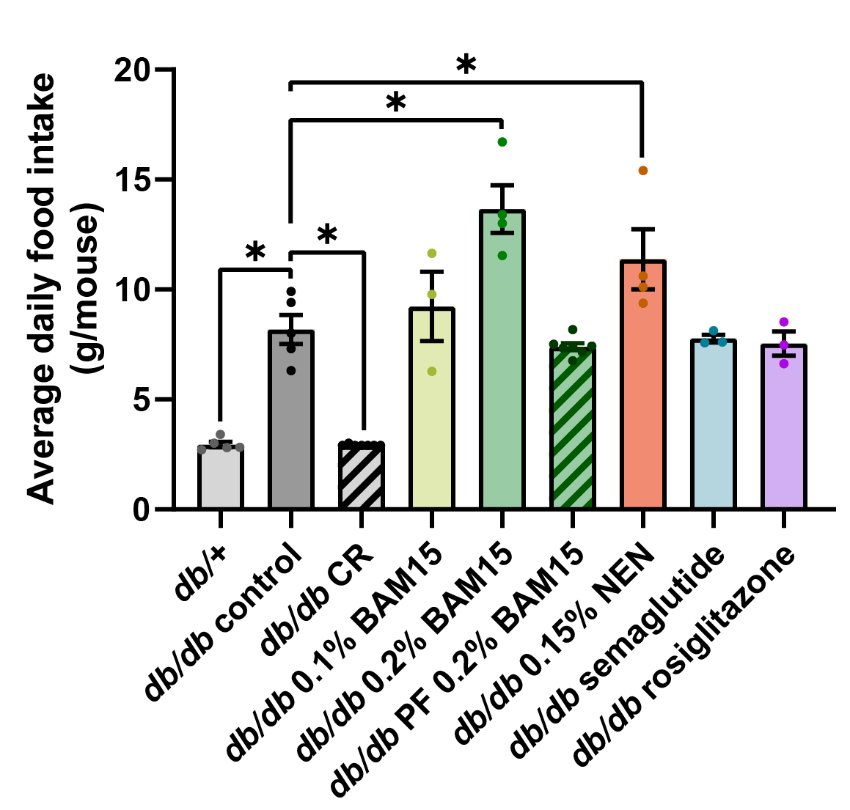


**Figure S1. Food intake in female *db/+* and drug-treated *db/db* mice.** Food intake was measured during the 4-week study for all treatment groups. Statistical significance was analysed by One-Way ANOVA with Tukey’s multiple comparisons test, comparing each group to every other group. Only significant changes (p<0.05) compared to *db/db* control are shown, indicated by *. Graph shows mean±SEM. n=3-7 cages per group.
